# Independent evaluation of a FOXM1-based quantitative malignancy diagnostic system (qMIDS) on head and neck squamous cell carcinomas

**DOI:** 10.18632/oncotarget.10512

**Published:** 2016-07-09

**Authors:** Hong Ma, Haiyan Dai, Xiaofeng Duan, Zhenglong Tang, Rui Liu, Kunjun Sun, Ke Zhou, Hao Chen, Hang Xiang, Jinsheng Wang, Qiong Gao, Yuan Zou, Hong Wan, Muy-Teck Teh

**Affiliations:** ^1^ China-British Joint Molecular Head and Neck Cancer Research Laboratory, Department of Oral and Maxillofacial Surgery, Hospital and School of Stomatology, Guizhou Medical University, Guizhou, China; ^2^ Institute of Dentistry, Barts and The London School of Medicine and Dentistry, Queen Mary University of London, England, United Kingdom

**Keywords:** molecular diagnostics, ethnicity, early cancer biomarkers, squamous cell carcinoma, FOXM1

## Abstract

The forkhead box M1 (FOXM1) transcription factor gene has been implicated in almost all human cancer types. It would be an ideal biomarker for cancer detection but, to date, its translation into a cancer diagnostic tool is yet to materialise. The quantitative Malignancy Index Diagnostic System (qMIDS) was the first FOXM1 oncogene-based diagnostic test developed for quantifying squamous cell carcinoma aggressiveness. The test was originally validated using head and neck squamous cell carcinomas (HNSCC) from European patients. The HNSCC gene expression signature across geographical and ethnic differences is unknown. This is the first study evaluated the FOXM1-based qMIDS test using HNSCC specimens donated by ethnic Chinese patients. We tested 50 Chinese HNSCC patients and 18 healthy subjects donated 68 tissues in total. qMIDS scores from the Chinese cohort were compared with the European datasets (*n* = 228). The median ± SD scores for the Chinese cohort were 1.13 ± 0.66, 4.02 ± 1.66 and 5.83 ± 3.13 in healthy oral tissues, adjacent tumour margin and HNSCC core tissue, respectively. Diagnostic test efficiency between the Chinese and European datasets was almost identical. Consistent with previous European data, qMIDS scores for HNSCC samples were not influenced by gender or age. The degree of HNSCC differentiation, clinical stage and lymphatic metastasis status were found to be correlated with qMIDS scores. This study provided the first evidence that the pathophysiology of HNSCC was molecularly indistinguishable between the Chinese and European specimens. The qMIDS test robustly quantifies a universal FOXM1-driven oncogenic program, at least in HNSCC, which transcends ethnicity, age, gender and geographic origins.

## INTRODUCTION

Head and neck squamous cell carcinoma (HNSCC) is affecting over half a million people worldwide each year [1]. Its 5-year survival rates are poor (16–29%) among patients with late stages (Stage IV and pN3) of HNSCC [2]. Human papilloma virus (HPV) associated HNSCC is known to have better prognosis compared to HPV-negative patients. However, the overall prevalence of HPV in HNSCC was found to be less than 25.9% [3]. According to the latest Cancer Statistics in China, there were approximately 135,100 new HNSCC cases and 70,700 deaths [4, 5]. Although HNSCC is not as common compared to esophageal cancer in China, whilst incidence and mortality rates of esophageal cancer are declining [6], HNSCC incidence and mortality rates are both increasing despite improvements in treatment modalities [4, 5]. Alarmingly, HNSCC mortality rate increases dramatically from age 35 to 85 by more than 65-fold despite only a moderate (11-fold) increase in incidence rate within this age range [4], emphasising an urgent need to identify and treat patients as early as possible. When comparing urban to rural areas in China, urban incidence rate was 40% higher than rural areas but no difference was found for mortality rates between the two areas [4], suggesting there may be a systemic problem in current diagnostic and/or treatment interventions that leads to no improvement in survival rates despite higher detection rates in the urban population. This is likely due to the inability to identify high-risk patients at early stages when treatment is most effective. The 5-yr survival for early localised cancers can exceed 80% but falls to less than 20% in late stage tumours especially when regional lymph nodes are involved [2]. Such data is neither surprising nor exclusive to China. A worldwide consensus opinion appears to be that of tumour heterogeneity hampering accurate diagnosis/prognostication which impacts on treatment insufficiency in turns lead to high rates of tumour recurrence and no improvement in survival rates over the last 3 decades [7–9]. Early treatment can significantly safe long-term costs and improve survival by avoiding expensive, invasive head and neck surgery which often leads to debilitating consequences not only affects feeding, speech and vision, but may also destroy the face, disrupting one's personal identity. It is well documented that improved diagnostic and prognostic accuracy to inform the most appropriate intervention could significantly improve patient outcome, reduce mortality and alleviate healthcare costs [10].

In 2013, we have developed a FOXM1-oncogene associated multi-biomarker ‘quantitative Malignancy Index Diagnostic System’ (qMIDS) [11] for quantifying the aggressiveness of squamous cell carcinoma (SCC). FOXM1 transcription factor has been shown to be amongst the top upregulated oncogenes across 39 cancer types and is a major predictor of poor cancer prognosis [12]. The qMIDS assay therefore represented the first FOXM1-based cancer diagnostic test which was previously validated on patients living in the UK and Norway [11]. Given that cancer is often heterogeneous, one marker alone would not be reliable or accurate for diagnosis. Hence, qMIDS was demonstrated previously to involve FOXM1 plus 13 FOXM1-associated genes (HOXA7, AURKA, NEK2, CCNB1, CEP55, CENPA, DNMT3B, DNMT1, HELLS, MAPK8, BMI1, ITGB1 and INV) as a panel of 14 biomarkers (and 2 reference genes) for quantitative diagnosis of malignancy [11]. We had previously shown that qMIDS test were able to quantitatively segregate between normal and malignancy whilst unaffected by non-malignant inflammatory condition (lichen planus). The present study was carried out to independently compare and evaluate the use of qMIDS assay for diagnosing HNSCC in non-European patients, for which we carried out a study in China involving ethnic Chinese. The qMIDS assay was also independently setup and performed in China to rule out bias and inherent technical factors.

## RESULTS

**Figure 1:**
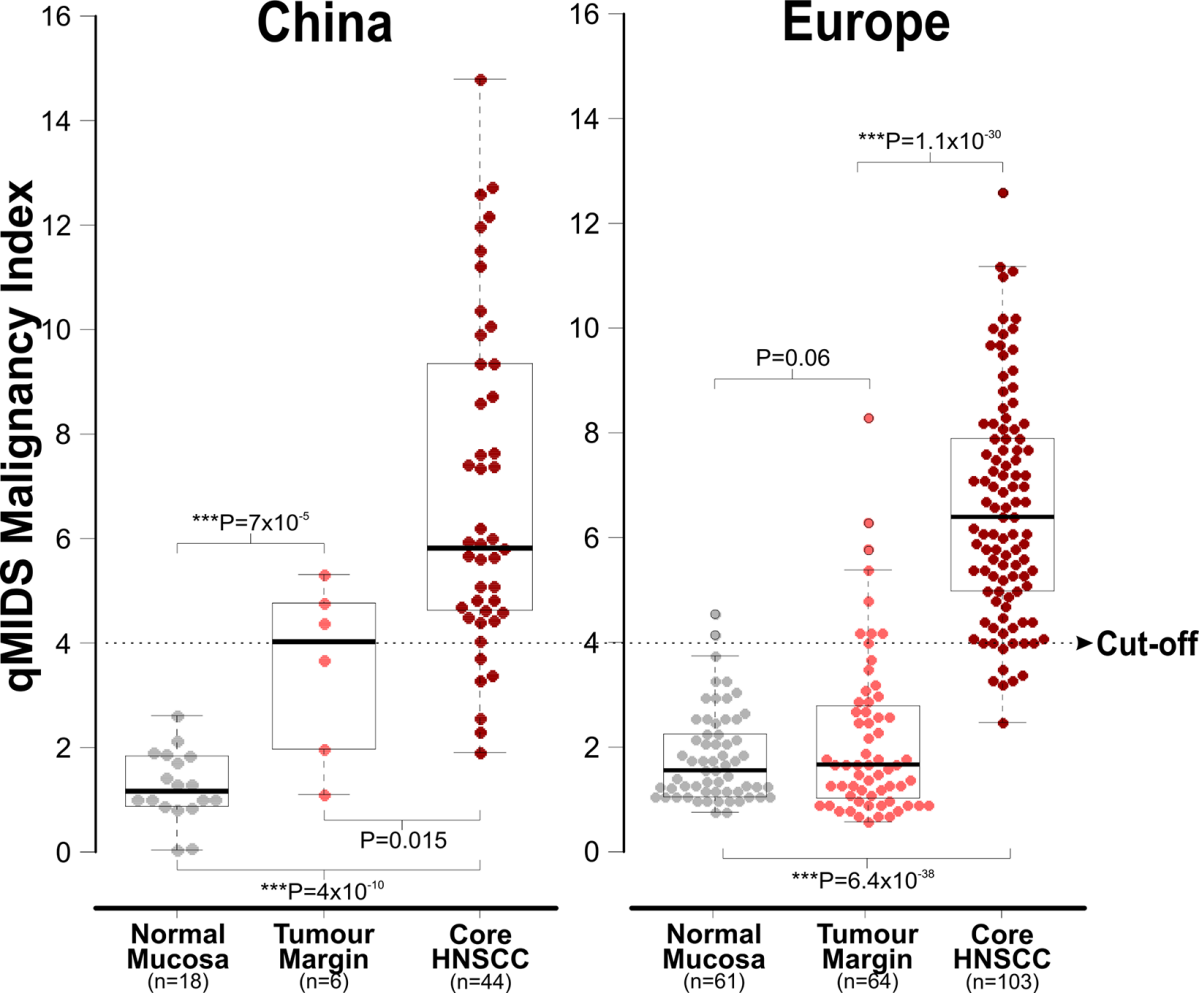
Comparison of qMIDS scores between Chinese and European head and neck tissue samples Data were plotted as dot-plot with box-and-whisker overlays (median and 25–75% percentiles). An optimum cut-off at 4.0 was found previously based on the European samples [11]. Statistical Student-*t* tests were performed between sample groups and corresponding *P* values were as indicated within the figure.

This study performed qMIDS assay on 68 Chinese head and neck tissue specimens. The normal group had 18 subjects donating normal oral mucosa tissues. The HNSCC patients donated 50 tumour core HNSCC tissues and 6 of these with additional adjacent tumour margin tissues. These tissues were originated from the tongue (*n* = 20, 45.5%), gingival (*n* = 8, 18.2%) buccal mucosa (*n* = 4, 9.1%), lip mucosa (*n* = 3, 6.8%), floor of mouth (*n* = 3, 6.8%), and other parts of the head and neck (*n* = 6, 13.6%). The median ± SD qMIDS scores for the Chinese cohort were 1.13 ± 0.66, 4.02 ± 1.66 and 5.83 ± 3.13 for healthy mucosa tissue, adjacent tumour margin and core HNSCC tumour tissue, respectively. For comparison, qMIDS scores were extracted from the European study [11] (dysplasia and lichen planus cohorts were excluded as these were not recruited in the Chinese cohort) whereby the median ± SD qMIDS scores were 1.50 ± 0.88, 1.70 ± 1.56 and 6.40 ± 2.11 for healthy mucosa tissues (Norway *n* = 61), adjacent tumour margins (UK, *n* = 64) and core HNSCC tumour tissue (UK, *n* = 103), respectively (Figure 1).

**Figure 2:**
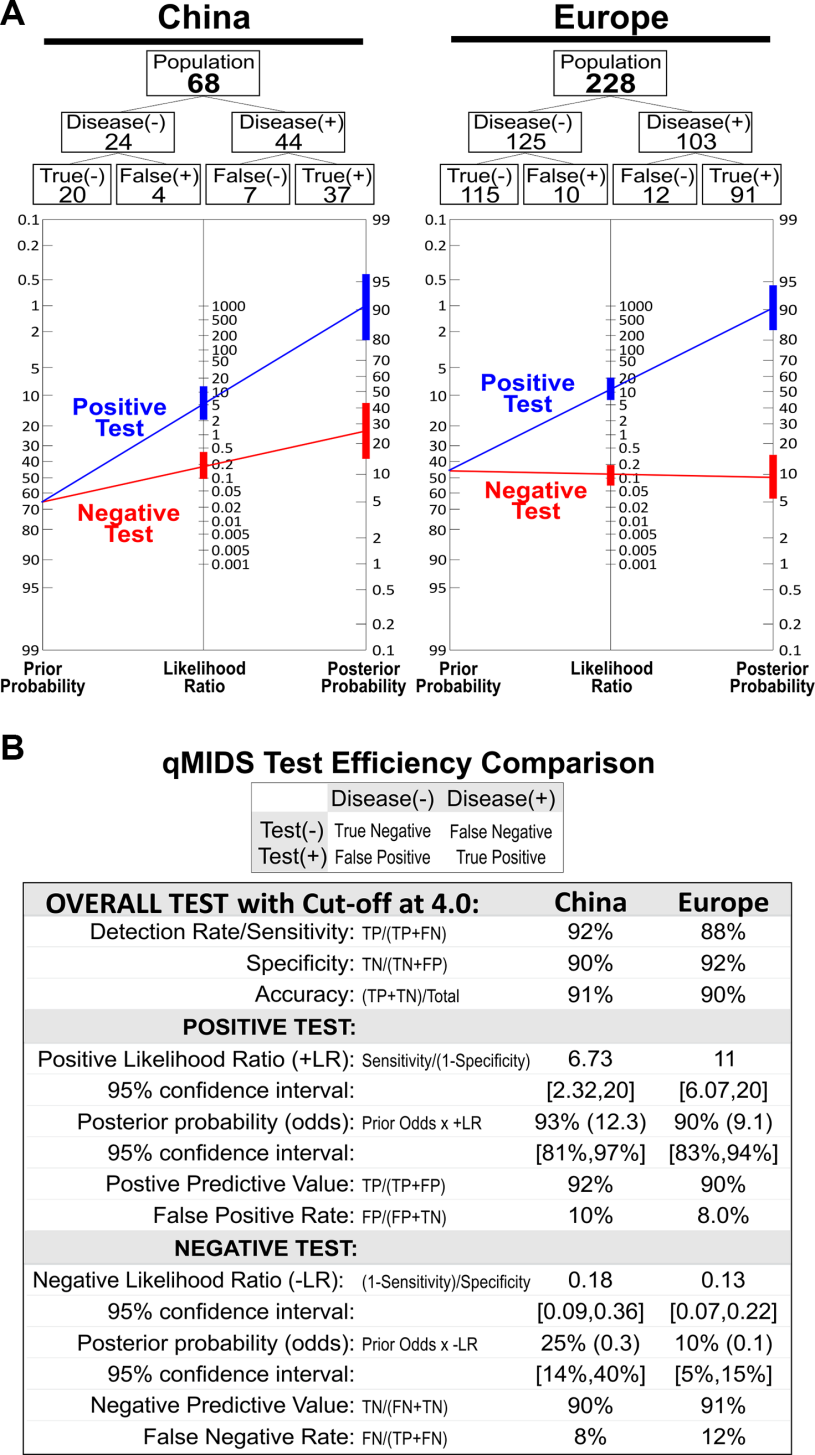
qMIDS Diagnostic test efficiency comparison between Chinese and European cohorts (**A**) Cohort analysis for Chinese (*n* = 68) and European (*n* = 228, consisting of UK and Norwegian participants, data were extracted from previous publication [11]). Calculations were based on cut-off score at 4.0 and statistical results are compared in panel (**B**).

The Chinese normal oral mucosa samples showed slightly lower qMIDS scores compared to the normal samples from Europe. Both the Chinese and European samples were showing highly significant segregation of qMIDS scores between normal and tumour samples, respectively. Unlike the European cohort, the Chinese adjacent tumour margin samples showed significant 2.4-fold higher scores when compared to the normal samples. Based on the previous European study [11], an optimum cut-off score value was at 4.0. This cut-off value was therefore used in the current study to calculate and compare the diagnostic test efficiency for qMIDS assay on the two cohorts (Figure 2A). The normal samples were grouped together with tumour margin samples as disease free group for the diagnostic test efficiency calculation. Overall, the diagnostic efficiency data between the Chinese and European cohorts was highly comparable (Figure 2B).

Further analysis of clinicopathological features within the Chinese HNSCC samples (*n* = 44), we found no differences between gender or age, which were in agreement with the European data. Statistically significant differences were found when HNSCC samples were segregated into differentiation status, tumour staging and lymphatic metastasis (Table 1). These findings were similar to previous European data whereby qMIDS scores were inversely correlated with differentiation status of HNSCC and were not significantly affected by gender and age [11]. We have previously established that HPV status did not affect qMIDS scores in neither HNSCC nor vulva SCC samples (data not shown) hence it was not further investigated. As habits such as smoking and drinking are well established as risk factors for HNSCC, due to the scarcity of patient records for risk factors, we were unable to analyse the correlation between habits and qMIDS scores.

**Table 1:** qMIDS scores and clinicopathological features of Chinese HNSCC (*n* = 44)

Clinical Features	Groups	*N*	Mean*	SD**	*t*	P***
**Gender**	Male Female	24 20	6.46 7.27	3.23 3.04	0.85	0.40
**Age**	< 60 > 60	13 31	6.95 6.78	2.86 3.28	0.17	0.87
**Differentiation Status**	High Moderate/Poor	32 12	5.44 10.55	1.96 2.61	7.04	**1 × 10^−8^**
**Tumour Staging**	I and II III and IV	35 9	5.88 10.55	2.39 3.01	4.89	**1 × 10^−5^**
**Lymphatic Metastasis**	No Yes	34 10	5.67 10.47	2.32 2.85	5.36	**3 × 10^−6^**

* Mean qMIDS score;

** SD = standard deviation;

*** P values in bold are highly significant P > 0.001.

## DISCUSSION

For many cancer types, especially HNSCC, tumour heterogeneity has been a key problem that eluded clinicians whereby histopathological findings could not provide a quantitative and objective correlation with tumour aggressiveness [8, 16]. To resolve this issue, we have previously developed a molecular method, the qMIDS assay [11], by exploiting the aberrant expression of a key oncogene FOXM1 shown to be amongst the top upregulated oncogenes across 39 cancer types and is a major predictor of poor cancer prognosis [12]. We and others have previously confirmed that FOXM1 is one of the top oncogene in HNSCC [11, 17–24]. We have previously published our bioinformatics meta-analysis on across over 40 different human cancer types available in Oncomine and NCBI's Gene Expression Omnibus (GEO) databases, showing that FOXM1 is one of the top oncogenes in HNSCC [18, 21].

Due to the heterogeneity found in many cancer types including HNSCC, using a single gene as a biomarker is unlikely to be accurate for quantifying tumour aggressiveness. To improve diagnostic accuracy and specificity, the qMIDS assay had been designed to quantify mRNA levels of 14 FOXM1-associated genes (HOXA7, AURKA, NEK2, FOXM1B, CCNB1, CEP55, CENPA, DNMT3B, DNMT1, HELLS, MAPK8, BMI1, ITGB1 and IVL) involved in the regulation of cell proliferation [25], differentiation [17], ageing [26], genomic instability [16, 18, 24, 27, 28], epigenetic [18, 20] and stem cell reprogramming [17, 29–31] as a collective basis to measure cancer aggressiveness via an algorithm to compute a malignancy index [11]. The qMIDS test was originally validated in the UK involving 256 Caucasian (from UK and Norway) and 36 South Asian (resided in the UK) patients. The assay was found to be a practical, sensitive, objective, and quantitative method for detecting not only for HNSCC, but also applicable for vulva and skin squamous cell carcinomas [11]. We had also previously shown in the Norwegian retrospective study with 19 years of HNSCC survival data that qMIDS score was significantly correlated with tumour aggressiveness [11] thereby providing a method for quantitative diagnosis and objective stratification of cancer aggressiveness.

Previous studies have reported that geographical, lifestyle and ethnic differences can impact on genetic/molecular pathways in head and neck squamous cancers [32–37]. Majority of these studies investigated genetic DNA polymorphisms but none of them, to our knowledge, compared gene expression levels in HNSCC. We are presenting the first study comparing different ethnic groups and gene expression levels in HNSCC using a FOXM1-based cancer diagnostic system [11]. Although the 14 genes used in the qMIDS assay were fundamental genes regulating squamous cell carcinoma, it was not clear if environmental factors (food, cultural & geographical variations, etc.) coupled with differences in ethnicity may impact on molecular differences in HNSCC that render the qMIDS test invalid. Given that the HNSCC patients tested previously constituted mainly of ethnic Caucasians (~86%) and South Asians (~14%) whereby all the patient samples were obtain either in the UK or Norway, we therefore aimed to further validate the qMIDS test to involve an entirely distinct ethnicity located in another geographic continent and have the assay independently set up and run in a different laboratory using different instruments (but using the same reagents). For this purpose, we recruited a total of 68 ethnic Chinese participants of whom, 50 were HNSCC patients and 18 were healthy individuals. All participants in this study were residence of Guizhou Province in China. The results obtained from this study on Chinese specimens were highly comparable to previously published European (UK and Norway) cohort [11]. Using the previously determined optimum cut-off score at 4.0 [11], overall diagnostic test efficiency was found to be almost identical between the Chinese and European datasets.

We have previously shown that the qMIDS assay had a detection rate of 90–94% and false positive rate of 1.3–3.2% on the European patients [11]. These data were consistent with the current study on Chinese patients. We had previously demonstrated that qMIDS was able to differentiate between benign (low risk) lesions such as oral lichen planus or fibro-epithelial polyps with premalignant (high risk) oral dysplastic samples [11], due to scarcity of Chinese patients with premalignant oral lesions (probably due to lack of self-awareness on oral diseases and patients were generally of lower social economic status), unfortunately we did not get sufficient number of these lesions for investigation. We are currently investigating the use of qMIDS as a tool for early oral premalignant cancer risk stratification.

In addition to HNSCC diagnosis, we previously demonstrated another clinical utility for qMIDS in tumour margin analysis whereby a 2D molecular topology of resolution down to 1 mm could be reconstructed using qMIDS on surgical samples. This was possible because each qMIDS test requires only a minute 1–2 mm tissue sample for analysis [11]. Although we did not carry out similar tumour margin analysis, the present study found a notable 2.4-fold higher qMIDS score in the Chinese adjacent tumour margin tissues compared to that of the European. This could be due to confounding factors such as error in pathological classification of the tissue samples and/or differences in width of surgical margins used. Although the difference was found to be statistically significant, the Chinese sample size was small (*n* = 6) and therefore caution in interpretation should be exercised here for the adjacent tumour margin group. Due to the sensitivity of qMIDS test, it is not surprising that some of these tumour margin samples did contain malignant cells that escaped detection by pathologists. Further study involving larger sample size with patient follow up may potentially reveal a relationship between qMIDS-positive tumour margins and tumour recurrence.

Similar to histopathology, qMIDS also involves testing tissue biopsy samples and hence it remains invasive and prone to mis-sampling issues. However, as field change is a common phenomenon in HNSCC [38–40] and that qMIDS detects molecular changes (mRNA expression) that precedes phenotypic change (protein and structural alterations), the sensitivity of detecting pathological genetic change in a given sample would arguably be much higher than that of histopathology which relies solely on visualising protein and structural change. Furthermore, dysplastic phenotype is often missed or misinterpreted when examining histopathological slides because molecular changes indicative of malignant conversion do not necessarily produce clinically or histopathologically detectable changes [38, 39]. Hence, given that qMIDS detects molecular changes, it would be more resistant to sampling issues (considering oral field changes) compared to histopathology.

Current clinicopathological features are unable to predict tumour aggressiveness [41–43]. As a result, current practise is that most patients with oral premalignant disorders (OPMD) are indiscriminately put on time consuming, costly and stressful surveillance [42, 43]. Such “waiting game” creates unnecessary anxiety and stress for majority (88%) of low risk patients whilst delaying and under-treating minority (12%) of high risk patients [44]. A systematic review estimated a malignancy conversion rate for OPMD is 12% [44]. Given 135,100 HNSCC cases in China each year [4], and 70% of HNSCC preceded by OPMDs [45], the estimated total number of OPMDs would therefore be over 788,000 cases/year. Most patients only return when tumours have grown to advance stages when it is difficult to treat or untreatable. Delayed treatment thereby directly causes poor long-term morbidity and survival [7, 8, 16, 42, 43]. The current lack of a ‘case-finding’ diagnostic test results in ineffective patient management and unnecessary long-term financial burden to both patients and healthcare establishments. With a molecular test such as qMIDS, we have shown promising results previously that qMIDS was able to detect malignant cells in otherwise clinicopathologically “normal-looking” biopsy tissue [11] and therefore we are currently investigating the clinical use of qMIDS for identification of premalignant lesions.

In summary, this study provided the first evidence that the pathophysiology of HNSCC was molecularly (at mRNA levels) very similar between the Chinese and European specimens. Furthermore, it reiterates that the qMIDS assay robustly measures a universal oncogenic program driven by FOXM1, at least in HNSCC, which transcends ethnicity, age, gender and geographic origins. A high throughput, cost-effective and robust test such as qMIDS may play an important role for quantitative diagnosis of ambiguous biopsy specimens and/or to provide an objective diagnosis based on digital molecular profile to avoid mis-diagnosis. Given that majority (88%) of oral lesions are benign [44], identifying 12% of high risk potentially malignant oral lesions is notoriously difficult [41–43]. Further study involving testing oral premalignant lesions with qMIDS and long-term correlation with follow-up study would enable the qMIDS test to be used as an early cancer test.

## MATERIALS AND METHODS

### Patient recruitment and study protocol

All 50 patients with HNSCC admitted from June 2014 to August 2015 were selected, 6 of these patients provided paired adjacent tumour margin and core HNSCC tumour specimens. In addition, 18 healthy individuals (undergone either wisdom tooth extraction or facial restorative/reconstruction surgery) donated redundant normal oral mucosa tissues for this study. All patients and healthy individuals in this study were ethically Chinese and natives of Guizhou Province in China. All clinical samples were collected according to local ethical committee-approved protocols and informed patient consent was obtained from all participants. The study was approved by the Institution Review Board of Human Ethics Committee of Guizhou Medical University. For each patient, histopathological reports of the tissue samples were obtained from collaborating clinicians. Fresh biopsy tissues were preserved in RNALater (#AM7022, Ambion, Applied Biosystems, Warrington, UK) and stored short term at 4°C (within 1 day) before transportation and subsequent storage at −80°C until use. All tissue samples were digested with nuclease-free proteinase K (Roche, UK) at 55–60°C before mRNA extraction (Dynabeads mRNA Direct kit, Invitrogen, UK) and cDNA synthesis (Transcriptor cDNA Synthesis kit, Roche, UK). All samples were tested blindly to ensure that the qMIDS assays were performed objectively.

### The qMIDS assay

The qMIDS assay methodology was described previously [11]. Briefly, the qMIDS assay involves quantification of mRNA levels of 14 target genes (HOXA7, AURKA, NEK2, FOXM1B, CCNB1, CEP55, CENPA, DNMT3B, DNMT1, HELLS, MAPK8, BMI1, ITGB1 and IVL) and 2 reference genes (YAP1 and POLR2A). We setup and run the qMIDS assay at our laboratory in Guiyang, School of Stomatology, Guizhou Medical University. In order to obtain data comparable to previous European data [11], we adhered tightly to the original qMIDS assay protocol for reverse transcription and quantitative PCR (qPCR) procedures as described previously [11]. qPCR reactions were setup in 96-well format (see supplementary Figure S1) and run on a Bio-Rad CFX Connect^TM^ Real Time System (Bio-Rad Life Science Research and Development Co., Ltd., Shanghai, China). Relative expression data for each target gene against the two reference genes were obtained using the Bio-Rad CFX manager 3.0 software. Relative expression data were then exported into Microsoft Excel for calculation of qMIDS score based on its original qMIDS algorithm [11]. Due to the tiny tissue size (1 mm^3^) used for each qMIDS assay and direct extraction of mRNA (rather than total RNA), quantification of mRNA yield was not accurate by neither spectrophotometer (eg., NanoDrop) nor fluorescence dye (eg., PicoGreen). Hence, data quality for each specimen was directly determined by qPCR based on the ability to measure both reference genes (YAP1 and POLR2A). Samples that failed one or both reference genes were omitted from the study.

### Statistical analysis

For comparison, qMIDS scores from the European study (data extracted from [11]) and the current Chinese data were analysed in R (version 2.13.1; The R Foundation for Statistical Computing) and plotted using Beeswarm Boxplot software package [13]. Diagnostic test performance between the European and Chinese data were compared at a specific qMIDS cut-off at 4.0 which was previously found to give the lowest false-positive rate and highest detection rate/sensitivity [11]. Diagnostic test efficiency comparison data were calculated using a Diagnostic Test Calculator freeware [14]. The qMIDS diagnostic assay efficiency tests were performed according to the STARD Initiative recommended protocol [15]. The qMIDS scores were also examined in relation to gender, age, differentiation status, tumour staging and lymphatic metastasis status, using the statistical package SPSS version 14.0. Kruskal-Wallis analysis was used to test the differentiation of the qMIDS scores among the three groups (normal mucosa, tumour margin and core HNSCC). The qMIDS scores of HNSCC were further examined using Student-*t* test for any relationships between the above mentioned clinical features using Student's test. *P* < 0.05 was considered statistically significant.

## SUPPLEMENTARY MATERIALS


