# Supplementary material for: Independent evaluation of a FOXM1-based quantitative malignancy diagnostic system (qMIDS) on head and neck squamous cell carcinomas
Source: Oncotarget. 2016 Jul 9;7(34):54555–63. doi: 10.18632/oncotarget.10512 (PMC5342363; doi:10.18632/oncotarget.10512)
Supplement: Supplementary file 1 [file oncotarget-07-54555-s001.pdf]

Independent evaluation of a FOXM1-based quantitative malignancy diagnostic system (qMIDS) on head and neck squamous cell carcinomas

Supplementary Materials

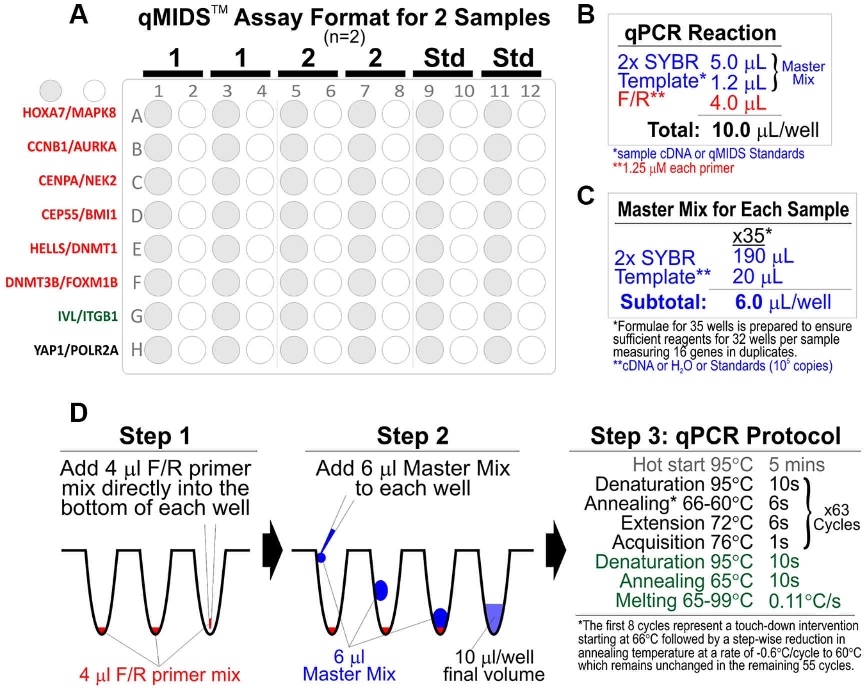

Supplementary Figure S1: qMIDS 96-well assay format and protocols. (**A**) qMIDS assay layout for 2 samples in duplicates. (**B**) qPCR reaction composition per well. qMIDS standards consist of a mixture of all 16 biomarker templates at fixed concentration to give a final concentration of 10^5 copies/well. (**C**) Master mix preparation for each sample. (**D**) Primer (Step 1) and master mix (Step 2) loading procedures, and qPCR cycling protocol (Step 3).
